# More than just life and death: advances in imaging and analysis for 3D-bioprinted tissues

**DOI:** 10.3389/fbioe.2025.1600077

**Published:** 2025-06-11

**Authors:** Erin R. Spiller, Daniela F. Duarte Campos

**Affiliations:** Bioprinting & Tissue Engineering Group, Center for Molecular Biology of Heidelberg University (ZMBH), Heidelberg, Germany

**Keywords:** machine learning, 3D bioprinting, CNN - convolutional neural network, 3D cell culture, image analysis, AI image analysis, light microscopy

## Abstract

3D bioprinting is a fast-growing field with applications in both microphysiological systems and tissue engineering. However, the qualifications and definitions of success for 3D-bioprinted products are insufficient. We can further our characterization of 3D-bioprinting methods and finished products using new imaging techniques and analysis methods, including the use of AI tools. This multi-faceted approach can deepen our understanding of valuable technology by examining the effects of 3D bioprinting on cell identity, behavior and organelles. Defining a successful 3D-bioprinted product in addition to viability is crucial in the push toward using these models for drug screening or disease modeling, where robust and high-quality systems are required for meaningful data output.

## Introduction

3D bioprinting is becoming more common within the bioengineering field, bringing many benefits. However, as with many nascent fields, validation and characterization tools need to develop simultaneously to continue robust advancement. With the rise in 3D-bioprinted constructs, some tools can be carried over from traditional cell culture while others may need to be adapted for use with 3D biological platforms. Early studies used viability as a criteria for success, which has remained the gold standard (Calvert, 2007; Strauss et al., 2023), typically, performed using a live/dead imaging assay to determine the cell viability in 3D-bioprinted models. However, more robust information is needed, as the bioprinting process can cause cell-damaging stress, for example, via shear stress, cytotoxicity, or phototoxicity (Deo et al., 2020). These stressors affect more than just cell viability; although live/dead assays provide important information, consideration must also be given to proliferation status, cell morphology, metabolic state, and cell lineage. Several imaging and analysis methods for 3D-bioprinted systems can provide insight into these features. As these are multicellular systems, the analysis methods must be able to handle large data sets with the potential segmentation of large numbers of cells. Analysis can be performed using several commercially available or open-source software tools. AI segmentation can speed up and automate analysis of these large data sets.

In traditional 2D cell culture, common assays used to characterize cells include vital dyes, metabolic assays, morphological analysis, and apoptosis/proliferation assays. Often, these characterizations are relatively straightforward for cells grown in a single layer. The addition of a third dimension adds some challenges to these characterizations–mainly lack of direct access to the cells and the bioinks’ opacity and permeability. Dyes and other reagents must penetrate the surrounding matrix to interact with the cells while the matrix’s transparency must be maintained for light-based microscopy. Inherent confounding factors also affect cell behavior such as oxygen and nutrient gradients from the surface of the structure to the center. We will discuss current light-based imaging and analysis practices within the bioprinting field as well as practices from other areas of cell culture that could be adapted for 3D cell culture.

## 3D-bioprinting methods

**FIGURE 1:**
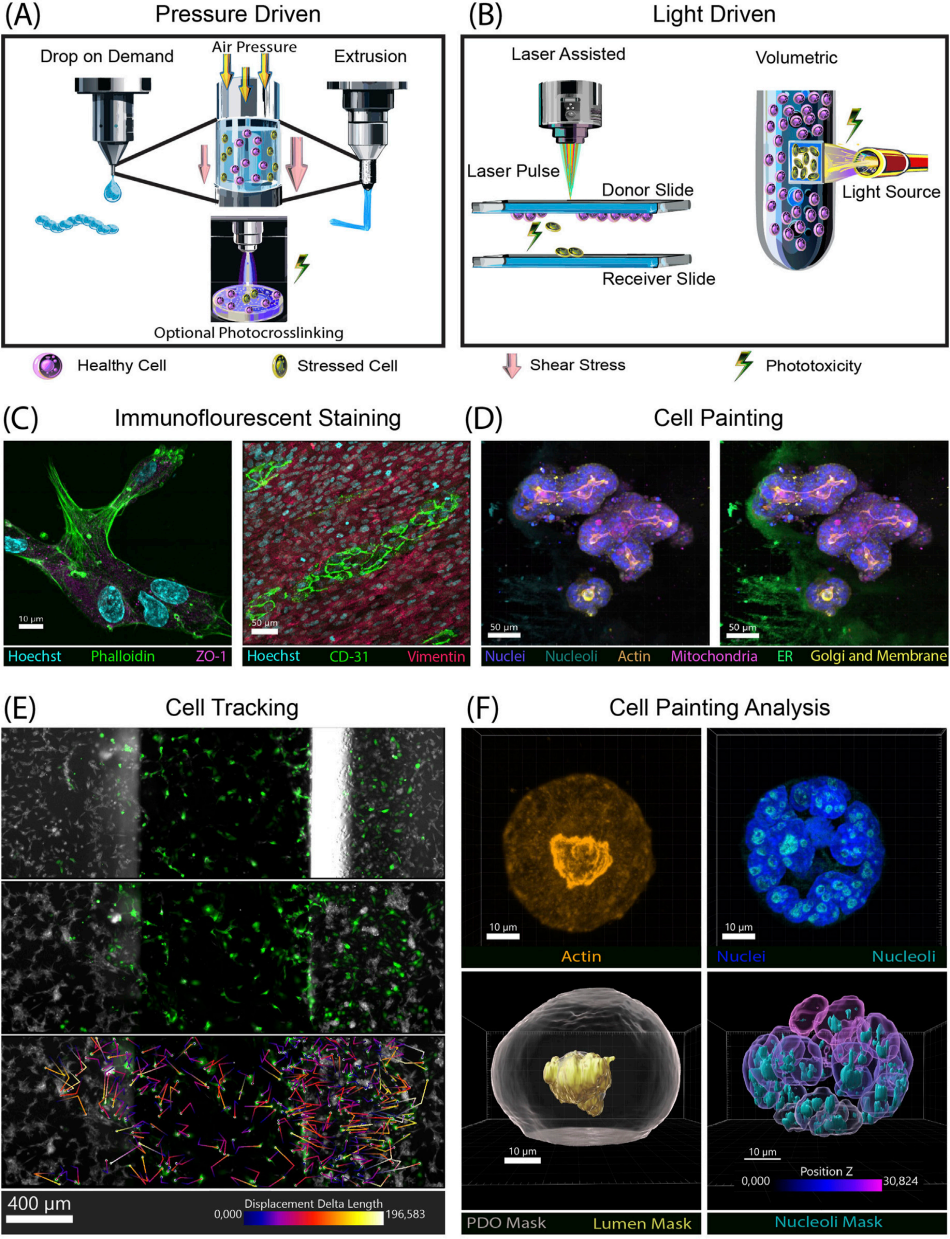
Bioprinting methods stress cells, examples of imaging and analysis tools to characterize bioprinted models. **(A)** Pressure based 3D biopringting methods, drop on demand (left) exerts less shear stress than extrusion (right). If the printing process includes photocrosslinking then phototoxicity is also possible. **(B)** Light based 3D bioprinting methods. Laser assisted (left) and volumetric (right) both rely on light to crosslink structures, leading to phototoxicity. **(C)** Immunofluorescent confocal images. (Left) Z-stack image of HUVECs encapsulated in BME (basement membrane extract) stained with phalloidin for actin (green), Hoechst for nuclei (cyan), and ZO-1 (magenta). (Right) Co-culture of cHIMECs stained with CD31 (green) and colon fibroblast stained with vimentin (red). Nuclei (Hoechst) are in cyan **(D)** Confocal z-stack image of cell painting on colon organoids embedded in BME. Nuclei (blue), nucleoli (cyan), mitochondria (magenta), actin (orange), golgi and membrane (yellow). Left, also shows ER (green) with off target staining of the BME. **(E)** Analysis of cell movement over in a chemotaxis chip over 2 days in µm. Multiple fields of view were stitched together to enable complete tracing in the x,y direction. Movement of GFP-HUVECs was tracked at three timepoints Day 0 (not shown), Day 1 (top), and Day 2 (middle). Color coded trajectories (µm) of each cell is show on the bottom panel. HCT116 cells (gray) stained with live cell tubulin **(F)** Z-stack images of a cell painted organoid. (Left top) actin (orange) (Right top), nuclei (blue) and nucleoli (cyan). (Bottom right) 3D rendered masks generated by Imaris with total organoid mask in gray, and lumen in orange. (Bottom right) nuclei color coded by z-location, mask of nucleoli in cyan. Antibodies used: Phalloidin-Alexaflour 488 (Thermo Fisher, A12379), ZO-1 (Abcam ab221547), CD31 (abcam, ab124432), Vimentin (abcam, ab20346), Phenoview cell painting kit (Revvity, PING11), PhenoVue Fluor 674 – Live Cell Tubulin (Revvity, CP21R1).

Printing methodologies can be categorized into pressure-based (Figure 1A) and light-based (Figure 1B) (Graham et al., 2017; Ozbolat and Hospodiuk, 2016; Murphy and Atala, 2014; Regehly et al., 2020). Each printing methodology uses cells encapsulated in compatible bioinks (natural or synthetic hydrogels) to create 3D structures and exerts various stresses on the cells that are not seen in traditional cell culture. It is vital to understand how the cell handling could affect the cells (i.e., shear stress in extrusion or droplet-based printing methods and phototoxicity in light-based methods). For example, shear stress can affect not only cell viability but also adhesion, proliferation, morphology and metabolic activity (Deo et al., 2020; Blaeser et al., 2016; Shi et al., 2018; Ng and Shkolnikov, 2024). In the cancer field, it has been shown that shear stress can cause epithelial-to-mesenchymal transition, a hallmark of cancer, and change gene expression (Choi et al., 2019; Alvarado-Estrada et al., 2021; Nauseef et al., 2012). Phototoxicity from UV or near-UV light can cause DNA damage, leading to carcinogenesis (de Gruijl et al., 2001; Wang et al., 2018). It therefore seems prudent for researchers to also qualify these factors compared to normal cell function, which can often be performed using a light-based microscopy method.

## Image-based evaluation methods

To evaluate viability, simple and readily available live/dead viability kits are often used (vital dyes such as Calcein AM/EthD-1). To understand the dynamics of both short- and long-term survival, viability is best evaluated at multiple points, as each will provide a single snapshot of both live and dead cell numbers (Zhang et al., 2019). Cell morphology dyes (e.g., phalloidin-rhodamine, CellTracker) pair nicely with viability stains (e.g., DRAQ7, thiazol-orange based DNA dyes) and can provide insight into cell type conformation or identification of morphological changes caused by the printing process (Zhai et al., 2018; Desroches et al., 2012; Chen et al., 2019; Akagi et al., 2013; Domahidy et al., 2024). Compared to monolayer, live cell dyes can present some challenges when used on 3D cultures. Monolayer or suspension cultures can accept dye into the media at any point, then perform a media change, removing residual background signal from unbound dye. Extracellular matrix (ECM) or bioink surrounding the cells can cause dye to stick in the bioink from binding or in pores within the matrix, which can create a high background signal during imaging or simply prevent the accumulation of dye in the cells resulting in a lack of signal. Mixing the dye before adding the bioink may produce a strong signal at the start of the experiment that decreases over time, causing challenges for long-term experiments. An alternative to tracking dyes is genetically engineered cells, which express a fluorescent protein. For example, H2B-GFP has a green fluorescent protein (GFP) fused to the histone-2b gene, resulting in GFP nuclei or cells that express a fluorescent protein in the cytoplasm (Kanda et al., 1998; Shagin et al., 2004; Shaner et al., 2004). Using fluorescent protein-expressing cells prevents interactions between the fluorophore and the ECM.

When examining dead cells, it could be beneficial to differentiate between apoptotic and necrotic cells. Differentiating live, apoptotic, and necrotic cells to determine the effect of shear stress during the printing process was performed using annexin-V, a marker of early apoptosis (Nair et al., 2009). Combining annexin-V with propidium iodide (PI) enables the identification of early apoptotic cells, allowing them to be differentiated from late apoptotic cells. Necrotic cells were defined as positive for both annexin-V and PI while apoptotic cells are those only positive for annexin-V. Cells negative for both are considered live. Other assays enable imaging of apoptotic cells over time using DEVD peptides conjugated to a nuclear dye. DEVD is cleaved by caspase 3/7 activating the fluorophore, enabling imaging detection of apoptotic cells (Cen et al., 2008). This method has been used in 3D with spheroids to determine drug response (Mittler et al., 2017). Currently, it is difficult to determine the number of cells that may go down the apoptotic path directly post bioprinting; tracking this process could make it easier to determine the bioprinting methods with the least amount of cell loss.

Immunofluorescent (IF) staining is a highly versatile method to examine many different aspects of a cell. Markers such as Ki67 can be used to determine proliferation while caspases can be used for cell death. This is important to differentiate since a viable cell may not be proliferating. Another use of IF antibodies is cell-specific markers, used to verify cell identity, and organelle markers that can be used to visualize cellular organelles. One group performed a differentiation assay to determine osteoblast phenotype over the course of 21 days (Zhai et al., 2018). Another group examined cellular differentiation status of cells grown on a 3D-printed scaffolding using lineage-specific markers (Chen et al., 2019). This same type of study could be transferred to bioprinted scaffolds. Specific antibody stains can be used to look at cell behaviors such as adhesion or cell junction formation to visualize a cell’s interaction with the surrounding matrix or cell-cell interactions (Figure 1C). Cell identity in a mixed culture can also be marked using cell-specific antibodies (Figure 1D).

Cell painting combines multiple fluorophores that stain specific individual organelles within cells. Originally developed for use in high-content, high-throughput screening, cell painting enables visualization of cellular response to perturbations (Seal et al., 2025). While this technique works best on cells grown in monolayer, our lab has had some initial success adapting cell painting to 3D-bioprinted cells and organoids (Figure 1E). One limitation has arisen: Concanavalin A can bind to the extracellular matrix surrounding the cells, which creates an artifact that limits the ability to perform analysis of images that include this dye. Further optimization and testing of a compound library could highlight its utility in the 3D-bioprinting field.

*In situ* analysis revealed increased proliferation on the periphery of printed GelMA constructs, which could be caused by multiple factors, including preparation, degree of functionalization, and cross-linking time. This exemplifies the importance of cell location when analyzing viability and proliferation (Allen et al., 2022). The location of cells within a matrix could affect the metabolic state due to oxygen and nutrient gradients. One way to understand this using imaging is to use fluorescent lifetime imaging (FLIM), measuring the decay time of endogenous fluorophores (e.g., NAD(P)H FAD) (Datta et al., 2020). Spatial metabolomics of 3D breast cancer spheroids and screening of cancer organoids for potential drug targets shows how this technique is already being used with 3D platforms (Karrobi et al., 2023; Tavakoli et al., 2025).

Epigenetic control of cell states via chromatin wrapped around histones is a mechanism of gene regulation in eukaryotic cells. Histone modification (e.g., methylation, acetylation) is known to regulate gene expression by modulating transcription factor access to promoters, thereby changing the production of genes, switching them on or off. Highly differentiated cells have a different histone modification pattern than cells that retain their stemness (Volker-Albert et al., 2020). As sheer stress can cause cell differentiation changing cell identity, especially in vascular cells, histone modification status could be of interest to determine if epigenetic changes are present (Wang et al., 2005). Acetylated and methylated histones can be visualized using fluorescently labeled antibody fragments (Fabs) in single cells (Hayashi-Takanaka et al., 2011). A recent advancement resulted in the development of Fab-based imaging of live-endogenous modifications (FabLEM) (Saxton et al., 2023), which was used to determine histone modifications and transcription initiation. This live cell imaging technique could help to answer questions surrounding the time dynamic of epigenetic changes post bioprinting.

## Analysis techniques

Image analysis can be performed for all techniques that use imaging as an output, turning qualitative data into quantitative data (Table 1). Many researchers achieve this using open-source ImageJ-based tools such as FIJI to quantitate their images (Schneider et al., 2012; Schindelin et al., 2012). Other open-source software includes CellProfiler for extracting quantitative date and CellProfiler Analyst to use machine learning (ML) algorithms to identify phenotypes (Stirling et al., 2021b; Stirling et al., 2021a). Advances in computer vision and ML now make it possible to quickly analyze large data sets. Cellpose is an open-source analysis tool with an algorithm that uses a neural network to segment both brightfield and fluorescent images (Stringer et al., 2021). Harmony software with Phenologic AI is a strong tool, but limited in its utility, as it is only compatible with Revvity (formerly PerkinElmer) imaging systems (Garvey et al., 2016). These programs can also provide morphological information in 3D systems such as spheroids and organoids (Spiller et al., 2021). Here, live/dead ML classification of organoids was compared to expert visual classification and DRAQ7. When all experts agreed on classification, concordance between ML and manual classification is 100%, yet only 62% between visual and DRAQ7, indicating morphological ML-based classification of organoids is more accurate than DRAQ7. Imaris (Bitplane) is another imaging analysis program focused on 3D images that enables the analysis of large data sets with a batch analysis feature. Using this software, dynamics of organoids and individual cells can be distinguished (Kim et al., 2020). Recently, Imaris software added machine learning algorithms for segmenting images based on images’ background and foreground classifications. However, the Imaris licensing cost can be prohibitive. Examples of analysis using Imaris, including cell tracking performed with time lapse imaging, are shown in Figure 1E. Imaging in 3D with multiple stains can provide multiple analysis of a single image (Figure 1F).

**TABLE 1:** Selected image analysis tools.

Analysis tools			
Program		Input images	Use Case example
Open source	FIJI	Brightfield, fluorescent	Segmentation
	CellProfiler	Brightfield, fluorescent	Segmentation, phenotyping, 3D
Commercial	Phenologic AI	Brightfield, fluorescent (only from own machine)	Segmentation, morphology, classification
	Imaris	Brightfield, fluorescent	Segmentation, tube formation, 3D
Convolutional Neural Networks	CellPose	Brightfield, fluorescent	Segmentation
	CeCILE	Brightfield	Radiation exposure
	Celldeath	Brightfield	Cell death
	Tellu	Brightfield	Organoid development and morphology

Convolutional neural networks (CNN) are a method of deep learning that consist of convolutional, pooling and fully connected layers (Li et al., 2022). U-Nets, named for their U-shaped architecture, are a type of CNN primarily used for image segmentation consisting of three main components: encoder, decoder, and a bridge to connect the two (Falk et al., 2019). The encoder extracts features, repeatedly applying convolutional layers and pooling them to reduce dimensionality while increasing the number of feature channels. The decoder up-samples the features previously mapped, gradually re-increasing the spatial resolution. The encoder concatenates the output, preserving fine details and improving segmentation.

Training of both CNNs and U-Nets is performed by manually labeling ground truth data, feeding it through the network before measuring loss (Dice loss, intersection over union). The network is then backpropagated with the loss measurements updating its weights. This process is repeated until output is satisfactory, and the algorithm is challenged with a validation data set to prevent over training. Input data are 2D stack slices; however, 3D analysis is possible with 3D convolutional filters applied to 3D input data (i.e., width, height, depth).

The image analysis field has recently seen many self-made algorithms based on machine learning applied directly to the creator’s area of interest. Cell Classification and *In-vitro* Lifecycle Evaluation (CeCILE), based on a CNN to detect radiation damage in cells (Rudigkeit et al., 2021), was trained on brightfield images of cells at different points in the cell cycle with and without radiation (mean f1_score_ = 0.93). Celldeath is another tool trained on brightfield images to detect cell death earlier than a human would be able to visualize these changes, with 97.23% test set accuracy vs. 50% manual classification accuracy (La Greca et al., 2021). Though both of those tools are applied to 2D cell culture, similar methods can be applied to 3D. Tellu, an open-source object detector and classification tool, is trained on brightfield images of organoids and can be used on movie files, not just single static images, with a 0.79 mean average precision (Domenech-Moreno et al., 2023).

Recently, Sheikh et al. used a CNN to determine spheroid viability, screening spheroids prior to use in 3D bioprinting and categorize them into four categories based on predicted viability (Sheikh et al., 2025). Their model has an accuracy of 92% with consistent loss below 0.2, demonstrating that these programs can be adapted to analyze 3D-bioprinted images. Algorithms that can both define regions of interest and sub-segment cells would be an optimal feature for analyzing 3D-bioprinted structures.

Cell polarization is also important to many tissues *in vivo*., as this ECM-driven process is vital to the functional structure of epithelial barriers (e.g., blood brain barrier, intestine), tissue development and repair (Lee and Streuli, 2014). This is simple to visualize with nuclear dyes, and the information can be obtained in parallel with other imaging assays by examining the directionality of nuclei in images. The Polarity-JaM tool suite could be used for this analysis (Giese et al., 2025) and demonstrates the advantage of answering multiple questions with a single imaging-based experiment. Images can be re-analyzed without having to perform additional wet lab experiments.

## Discussion

As the 3D bioprinting field continues to evolve, keeping some additional considerations in mind will ensure the quality and proper function of models. Live/dead staining is an easily accessible and valuable tool but does not provide the in-depth data necessary for a robust characterization. Differentiating between apoptosis (caused by normal cell turnover) and necrosis (triggered by cell injury) is vital to the understanding of cell behavior post printing. Although normal cell turnover should be expected over time, a missing mechanism for clearing cellular debris may cause other effects. Insufficient clearance of dying cells within an organism can cause persistent inflammation and tissue injury and lead to many chronic conditions in humans, including lupus and chronic obstructive pulmonary disease (COPD) (Krysko et al., 2008). As many bioprinted tissue constructs do not contain phagocytes, some abnormalities related to continued presence of dead and dying cells could contribute to further damage of otherwise healthy cells within the sample. Some of the imaging assays discussed here could be used to determine if this scenario lowers long-term survival of 3D-bioprinted models.

Of course, there are still many challenges to be solved when transitioning methods from 2D culture to 3D. One such challenge is analyzing intact samples, effected by both reagent and technical limitations (dye penetration, focal depth). If *in situ* information is needed, such as location-dependent cell survival rates, the construct must be analyzed intact. This could be significant in determining whether cell death is caused by oxygen and nutrient deficiency due to cellular location rather than the printing process itself. Often, imaging larger samples is challenging because they become more opaque, resulting in light scattering. Z-resolution is another confounding factor, as resolution is lost in thicker samples. Compensating for this by switching to super-resolution microscopy (e.g., fluctuation-based) requires a costly and specialized set-up. Alternatively, deconvolution of images can help to sharpen images by assigning pixel intensity to a single slice in a z-stack.

Progression in the 3D-bioprinting field needs innovative tools that help us to understand the systems being printed. To standardize MPS quality, there has recently been a call to develop much-needed standards similar to ISO criteria in other fields (Pamies et al., 2024). As we have shown here, a myriad of imaging-based assays and analysis tools could be used or adapted to further develop 3D bioprinting. Though the upfront investment for this pipeline is substantial (e.g., cost of equipment, commercial software), the return on investment could be high in industrial settings. On a smaller scale, academic settings often provide core facilities with specialized equipment and computing clusters for computational need. Expanding our understanding of 3D-bioprinted systems will benefit the field by facilitating production of high-quality model systems that consistently generate meaningful data.

## Data Availability

The original contributions presented in the study are included in the article/supplementary material, further inquiries can be directed to the corresponding author.
